# Clade-specific multiplex digital PCR assay for monkeypox virus detection in spike-in wastewater samples

**DOI:** 10.1099/acmi.0.001041.v3

**Published:** 2026-08-20

**Authors:** Melissa Pitton, Charles Gan, Patrick Schmidhalter, Christoph Ort, Timothy R. Julian

**Affiliations:** 1 Eawag, Swiss Federal Institute of Aquatic Science and Technology, Dübendorf, Switzerland; 2 Swiss Tropical and Public Health Institute, 4123 Allschwil, Switzerland; 3 University of Basel, 4055 Basel, Switzerland

**Keywords:** digital PCR, clade detection, monkeypox virus, mpox, multiplexing, wastewater epidemiology, wastewater surveillance

## Abstract

Monkeypox virus (MPXV) is a zoonotic pathogen that has recently caused outbreaks in non-endemic areas. Wastewater-based surveillance is independent of individual testing and offers substantial potential for monitoring population-level disease dynamics of MPXV at the clade level, as clades differ in transmissibility and mortality rate. Here, we report the validation of a four-plex digital PCR assay to detect and quantify the major clades and subclades of MPXV. The assay demonstrated strong linearity, analytical sensitivity (limits of detection ranged from 6.7 to 9.6 copies per reaction across targets) and specificity in distinguishing and quantifying MPXV by clade, which is advantageous for both clinical and wastewater applications.

## Data Summary

All digital PCR data presented in this study are available at: https://doi.org/10.25678/000GRJ.

## Introduction

Mpox is a zoonotic disease caused by the monkeypox virus (MPXV), which was first identified in humans in 1970. Since then, mpox has been endemic in Central and West Africa. MPXV is a linear double-stranded DNA virus belonging to the genus *Orthopoxvirus*. Two different clades of MPXV have been described until now, 1 and 2, which are both further classified into subclades (i.e. 1a, 1b, 2a and 2b). Clades and subclades can be distinguished molecularly by targeting gene variations or deletions; in this study, we used the tumour necrosis factor receptor gene (G2R) and the complement binding protein gene (C3L) targets [1]. Recent outbreaks in non-endemic regions have raised multiple concerns. Indeed, the World Health Organization (WHO) declared mpox a Public Health Emergency of International Concern twice within a 2-year period (2022–2024), which further highlights the importance of monitoring this pathogen.

Diagnostics of MPXV surveillance rely on PCR-based assays for detection and differentiation of MPXV clades. The use of multiplexed quantitative PCR (qPCR)-based assays provides quantitative data on multiple targets when analysed alongside paired standard curves representing known concentrations and semi-quantitative data when reporting cycle threshold values alone [2]. Digital PCR (dPCR) is an alternative to qPCR that provides quantification without the need for standard curves, although reference materials are still required for assay validation and optimization. Quantification of MPXV is relevant for assessing viral loads in clinical samples, which can be used to inform disease severity or transmission potential [3, 4]. Quantification is also important for MPXV surveillance within wastewater, which can lead to pathogen detection and improve knowledge about disease dynamics at the population level over time [5–11]. Importantly, the interim guidance document published by the WHO highlights the potential for wastewater-based surveillance (WBS) to provide actionable information on early detection, differentiation of circulating clades and quantification of viral loads in sewers, which can be used as an indicator for population-level disease dynamics [12].

A dPCR assay that simultaneously allows for clade differentiation is specifically important for WBS, as it can track trends and further highlight the relative proportions of multiple circulating clades within a community. In this study, we adapted four previously published assays to our droplet dPCR platform and then merged the individual assays to develop a four-plex assay for detecting and simultaneously differentiating MPXV clades. The developed assay is intended for applications that benefit from detection and quantification of individual MPXV clades in clinical or wastewater samples.

## Methods

### Experimental design

Four distinct independent assays were first adapted to the Bio-Rad QX600 AutoDG Droplet Digital PCR System (USA). These single-plex assays were previously described and validated: G2R_G assay, which targets all clades of MPXV (pan-MPXV) [1]; G2R_WA assay, which is specific to all clade 2 strains (MPXV-Clade2) [1]; C3L assay that specifically targets clade 1 a (MPXV-Clade1a) [1]; and dD14-16 assay, which targets the recently emerged clade 1b (MPXV-Clade1b) [13]. A four-plex was subsequently developed by combining the individual single-plex assays. The multiplex assay was validated through assessment of its analytical sensitivity and specificity. To demonstrate its applicability for wastewater-based surveillance, the four-plex assay was then evaluated using spiked wastewater extracts as a proof of concept.

### Positive controls

Assays were validated using positive controls derived from extracted genomes of clinical samples. DNA from MPXV clade 1a and 1b was provided by the WHO BioHub System, while MPXV clade 2b genomic DNA was obtained from the Public Health Laboratory of GGD Amsterdam.

### Droplet digital PCR assays

Primers and probes are listed and described in Table S1 (available in the online Supplementary Material) and were manufactured by Microsynth AG (Switzerland). The assays were developed on the Bio-Rad AutoDG Droplet Digital PCR (ddPCR) System (USA). All assays were prepared using a total of 22 µl pre-reaction volume, which consisted of 5.5 µl of template and 16.5 µl of mastermix. The mastermix was prepared according to the following procedure: ddPCR Supermix for Probes (No dUTP) (2×) (Cat. No. 1863024, Bio-Rad), 0.9 µM of each forward and reverse primer, 0.25 µM of each probe and RNase-free water. Thermocycling was performed using the following conditions: initial enzyme activation (95 °C for 10 min), 45 cycles of denaturation (95 °C for 15 s) and annealing/extension (60 °C for 1 min), followed by enzyme inactivation (98 °C for 10 min), and a hold (4 °C for 30 min). Droplet dPCR data were analysed using QX Manager 2.4 Standard Edition (Bio-Rad). Positive and negative droplet clusters were differentiated by applying a manually set threshold on one-dimensional amplitude plots (Fig. S1). Thresholds were positioned midway through the ‘rain’, defined as droplets exhibiting intermediate fluorescence amplitudes.

### Validation of the MPXV four-plex ddPCR assay

The MPXV four-plex assay was validated against the corresponding single-plex assays by measuring positive controls in triplicate at three concentration levels. A mixed stock solution containing all targets (i.e. extracted DNA from clades 1 a, 1b and 2b) was prepared by combining them to achieve a final concentration of ~300 copies per microlitre in the reaction and was subsequently subjected to tenfold serial dilutions in RNase-free water. For the multiplex assay, the positive control mix was analysed in a single reaction with the complete four-plex primers/probe mix. Agreement between the multiplex and single-plex formats was assessed using linear regression analysis. In addition, two-way ANOVA was performed to evaluate whether any observed differences between assay formats were statistically significant. The MPXV four-plex assay was further characterized by calculating the mean separation score [14], defined as the difference between mean fluorescence amplitudes of the positive and negative droplet populations divided by the sum of their respective standard deviations.

### Assay specificity and sensitivity

Assay specificity was evaluated by testing each positive control separately using the MPXV four-plex assay, to evaluate whether each positive control was detected by the pan-MPXV primer/probe set as well as by, and only by, the primer/probe set corresponding to its known clade. Reactions were performed in technical duplicates at three concentrations, each differing by a twofold dilution. Analytical sensitivity was assessed by determining the limit of detection (LoD), defined as the concentration corresponding to a 95% probability of detection. A twofold serial dilution consisting of 7 low concentrations was prepared, and 12 replicates were analysed for each concentration. Results were classified as ‘detected’ or ‘not detected’ based on a threshold of ≥3 positive droplets per reaction. A two-parameter Weibull distribution was then fitted to estimate the LoD for each target [15].

### Spike-in experiments in wastewater extracts

To evaluate whether the wastewater matrix affects mean separation scores, spike-in experiments were conducted. Positive control material, consisting of extracted DNA, was added to wastewater extracts previously collected and archived within the Swiss Wastewater Monitoring Program biobank [16]. The wastewater extracts (80 µl) originated from 24-h composite influent raw wastewater samples and had been processed using the Wizard Enviro Total Nucleic Acid Kit (Promega Corporation, USA), following a protocol with modifications as described previously [17]. PCR inhibition was calculated as previously described and was considered acceptable when it was below the 40% threshold [16].

## Results

### Adapting MPXV assays to our ddPCR platform

Previously published single-plex assays were adapted to our ddPCR platform, and each target had a specific fluorophore: FAM for pan-MPXV, HEX for MPXV-Clade2, ROX for MPXV-Clade1a and Cy5 for MPXV-Clade1b. We assessed linearity for each assay by measuring dilution series of positive material in technical triplicate. Our data showed strong linearity for each assay, with slopes of linear regression of 1, and *R*^2^ values of 1 (Fig. S2).

### Development and validation of the MPXV four-plex ddPCR assay

**Fig. 1.**
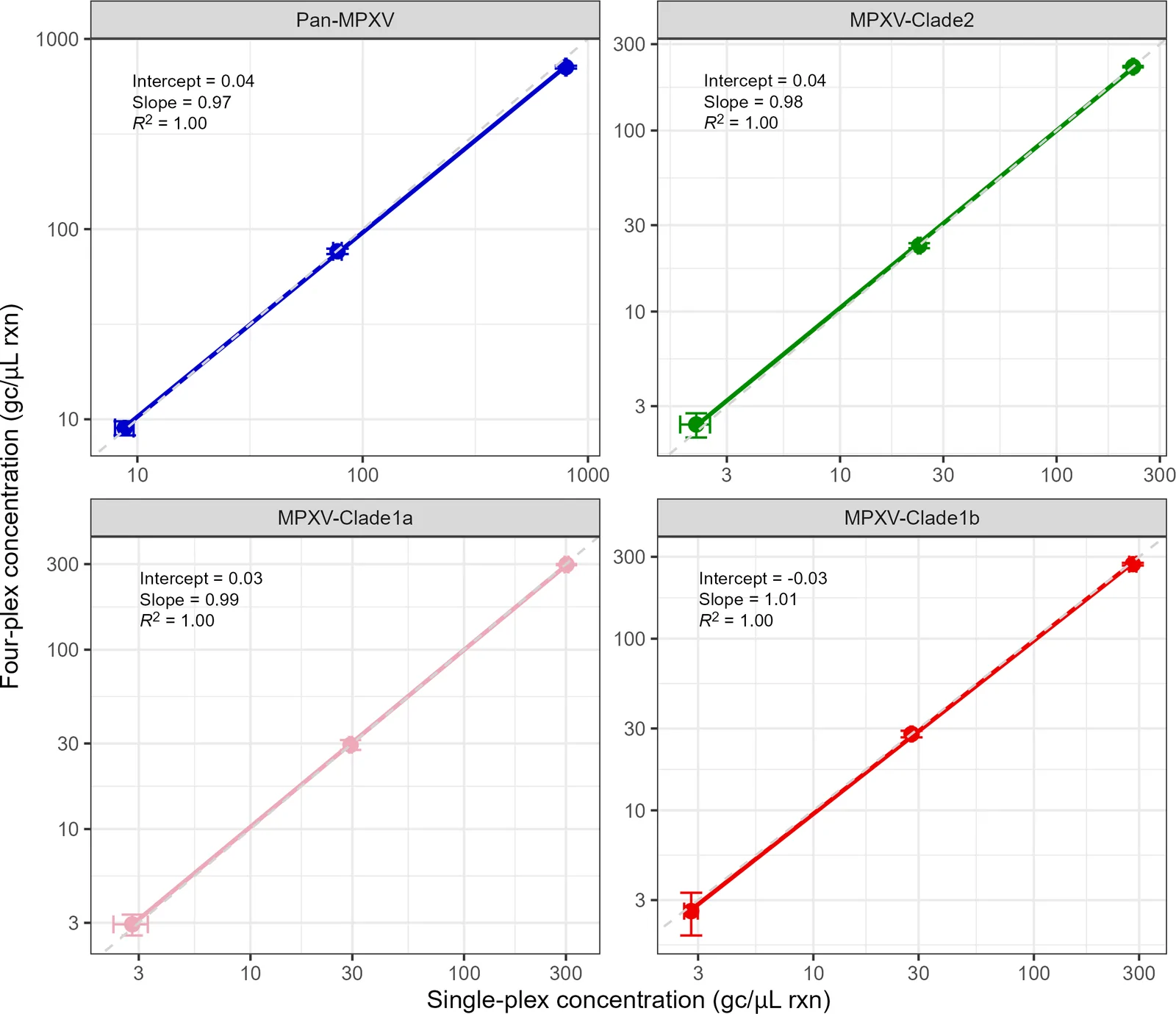
Linear regression of target concentrations measured by the four-plex ddPCR assay versus the corresponding single-plex assays. Each panel represents a specific target. Concentrations are expressed in genome copies per microlitre of reaction (gc/µl rxn) on a log_10_ scale. The grey dashed lines represent the identity lines, and coloured lines show the linear regression for each target. Error bars indicate the standard deviation.

We developed the four-plex assay by merging the individual assays. The performance of the novel four-plex assay was compared – in parallel – to that of the single-plex assays. Linear regression analysis demonstrated strong agreement between the assay formats, with slopes ranging from 0.97 to 1.01 and *R*^2^ values of 1.0 (Fig. 1). Our findings showed that all of the clade-specific assays exhibited no significant differences in quantification, as determined by two-way ANOVA (*P*=0.95 for MPXV-Clade2, *P*=0.49 for MPXV-Clade1a and *P*=0.20 for MPXV-Clade1b). The pan-MPXV assay detected all clades and expectedly estimated a value close to the sum of the clade-specific targets. Importantly, all positive droplet clusters from the targets were clearly distinguishable, allowing for differentiation between positive and negative partitions, as well as between distinct target genes (Fig. S1). Specifically, mean separation scores were well above the benchmark of 2.0–2.5 for all targets [18], with 9.3 for pan-MPXV, 5.8 for MPXV-Clade2, 13.6 for MPXV-Clade1a and 24.0 for MPXV-Clade1b.

### Sensitivity and specificity of the MPXV four-plex ddPCR assay

**Fig. 2.**
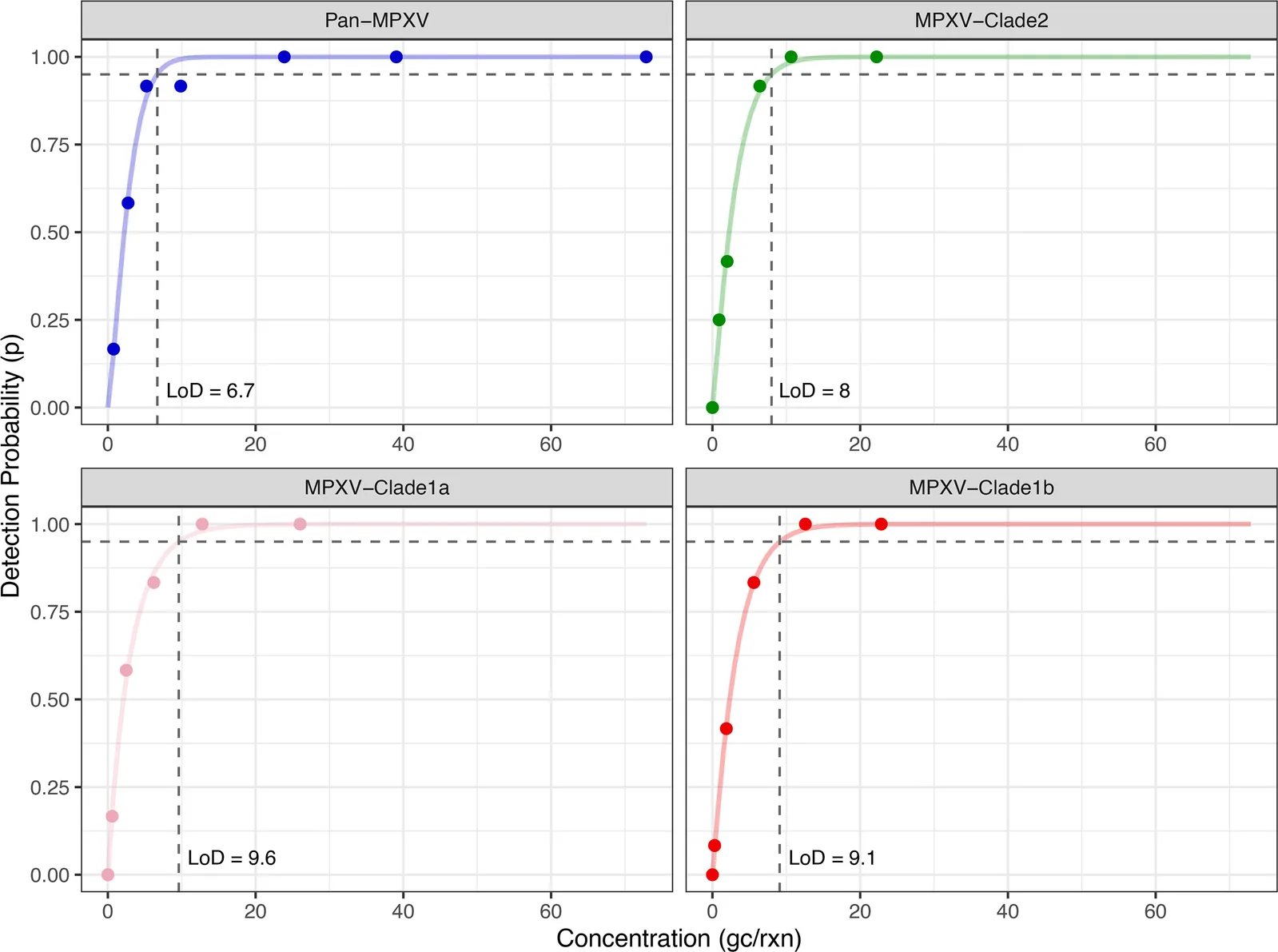
Analytical sensitivity of the newly developed ddPCR assay: limits of detection. Each panel shows a specific target. Coloured curves were modelled using a two-parameter Weibull distribution to fit the detection probability (**p**) across seven concentrations, expressed in genome copies per reaction (gc/rxn) and shown as coloured dots. Horizontal dashed line indicates *P*=0.95, corresponding to a 95% likelihood of detection, and the vertical dashed line indicates the LoD, defined as the concentration at which there is a 95% likelihood of detection.

The analytical sensitivity of the assay was assessed by testing seven low concentrations, ranging from 30 to 0.47 gc per reaction, each measured across 12 replicates. The LoD was subsequently determined by fitting a two-parameter Weibull distribution. The LoD at 95% confidence was 6.7 gc per reaction for pan-MPXV, 8 gc per reaction for MPXV-Clade2, 9.6 gc per reaction for MPXV-Clade1a and 9.1 gc per reaction for MPXV-Clade1b (Fig. 2). To determine the assay’s specificity, individual positive reference samples from different clades were tested. All samples were positive for pan-MPXV and their respective clade-specific targets and negative for the other targets, highlighting that the assay could reliably differentiate clades across a range of low concentrations (Fig. S3).

### Proof of concept for wastewater surveillance

Mpox cases have been reported only sporadically in Switzerland since autumn 2022 [19]. Therefore, the assay could not be tested directly on wastewater samples. However, as proof of concept, extracted DNA material was spiked into wastewater extracts to determine whether inhibition on PCR amplification is present, and cluster separation is impacted by the wastewater matrix. PCR inhibition was measured as previously described [17], and our analysis showed that no inhibition was found for MPXV-Clade1a and MPXV-Clade1b, and a mean inhibition of 0.5% for pan-MPXV and 7.8% for MPXV-Clade2 was observed, which is below our threshold for considering a sample as inhibited (i.e. 40%). Moreover, we observed that the wastewater matrix does not impair the separation score, which describes the discrimination between positive and negative partitions (Fig. S4).

## Discussion and conclusion

The application of PCR-based technologies has been proven useful for monitoring MPXV outbreaks [11, 20]. However – in the context of WBS – where different subclades can co-circulate within a community [21], various individual PCR assays need to be implemented for specifically differentiating clades of MPXV. Here, we report the development and validation of a four-plex ddPCR assay, which can detect MPXV and differentiate clade 1a and 1b and clade 2.

The assay demonstrated strong specificity and high analytical sensitivity, enabling reliable differentiation of clades even at very low concentrations. The four-plex ddPCR assay achieved LoD of 6.7 genome copies per reaction (gc per reaction) for pan-MPXV, 9.6 for MPXV-Clade1a, 9.1 for MPXV-Clade1b and 8.0 for MPXV-Clade2. These values are similar to or lower than values reported for previously published assays. For example, the MPXV-Clade1a LoD is markedly lower than the 40.4 gc per reaction reported by Li *et al*., while the MPXV-Clade2 LoD is comparable to their 8.2 gc per reaction [1]. Relative to the more recent evaluation by Scheule *et al*. [13], our assay shows lower LoDs for MPXV-Clade1 (9.6 vs. 11.4) and MPXV-Clade2 (8.0 vs. 59.9), while the MPXV-Clade2b sensitivity is slightly higher than their reported 6.6 gc per reaction. These differences likely reflect methodological variation between platforms, as ddPCR determines target quantity through reaction partitioning and Poisson-based calculations rather than standard curves. Nevertheless, the comparable sensitivities across studies demonstrate that previously validated single-plex assays can be successfully consolidated into a multiplex ddPCR format while maintaining strong analytical sensitivity and specificity.

Digital PCR technology offers absolute quantification of targets without the need for standard curves, which makes it appealing for both wastewater and clinical settings [22], and the multiplexing ability of this assay allows for the simultaneous detection and quantification of multiple circulating clades.

Our study was limited by the absence of MPXV circulation in Swiss wastewater, which prevented the possibility of testing the assay directly on wastewater extracts. However, spiking experiments – performed to mimic wastewater surveillance – showed that the wastewater matrix would not impact the assay’s efficiency, suggesting that the multiplex ddPCR assay could be readily implemented in the context of WBS.

## Supplementary material

10.1099/acmi.0.001041.v3 Supplementary Material 1.

## Abbreviations

dPCR: digital PCR
LoD: limit of detection
MPXV: monkeypox virus
qPCR: quantitative PCR
WBS: wastewater-based surveillance
WHO: World Health Organization
