# Supplementary material for: Clade-specific multiplex digital PCR assay for monkeypox virus detection in spike-in wastewater samples
Source: Access Microbiol. 2026 Aug 20;8(8):001041.v3. doi: 10.1099/acmi.0.001041.v3 (PMC13492878; doi:10.1099/acmi.0.001041.v3)
Supplement: Supplementary file 1 [file acmi-8-01041-s001.pdf]

Supplementary Material

Clade-specific multiplex digital PCR assay for monkeypox virus detection in spike-in wastewater samples

Melissa Pitton[1], Charles Gan[1], Patrick Schmidhalter[1], Christoph Ort[1], Timothy R. Julian[1,2,3,#]

1) Eawag, Swiss Federal Institute of Aquatic Science and Technology, Dübendorf, Switzerland.
2) Swiss Tropical and Public Health Institute, 4123 Allschwil, Switzerland.
3) University of Basel, 4055 Basel, Switzerland.

[#] Corresponding author: Timothy R. Julian, email: tim.julian@eawag.ch

Supplementary Table S1. Primers and probes used in the single-plex and MPXV four-plex assays.

Assay	Targeted region	Oligonucleotide name	Sequence (5' to 3')	Fluorophore/Quencher	Type	Reference
Pan-MPXV	G2R_G	G2R_G_F	GGAAAATGTAAAGACAACGAATACAG		Forward primer	Li et al
		G2R_G _R	GCTATCACATAATCTGGAAGCGTA		Reverse primer	Li et al
		G2R_G _P	AAGCCGTAATCTATGTTGTCTATCGTGTCC	FAM/BHQ-1	Probe	Li et al
MPXV-Clade1a	C3L	C3L_F	TGTCTACCTGGATACAGAAAGCAA		Forward primer	Li et al
		C3L_R	GGCATCTCCGTTTAATACATTGAT		Reverse primer	Li et al
		C3L_P	CCCATATATGCTAAATGTACCGGTACCGGA	ROX/BHQ-2	Probe	Li et al
MPXV-Clade1b	dD14-16	dD14-16_F	AAGACTTCCAAACTTAATCACTCCT		Forward primer	Schuele et al.
		dD14-16_R	CGTTTGATATAGGATGTGGACATTT		Reverse primer	Schuele et al.
		dD14-16_P	ATATTCAGGCGCATATCCACCCACGT	Cy5/BHQ-2	Probe	Schuele et al.
MPXV-Clade2	G2R_WA	G2R_WA_F	CACACCGTCTCTTCCACAGA		Forward primer	Li et al
		G2R_WA_R	GATACAGGTTAATTTCCACATCG		Reverse primer	Li et al
		G2R_WA_P	AACCCGTCGTAACCAGCAATACATTT	HEX/BHQ-1	Probe	Li et al

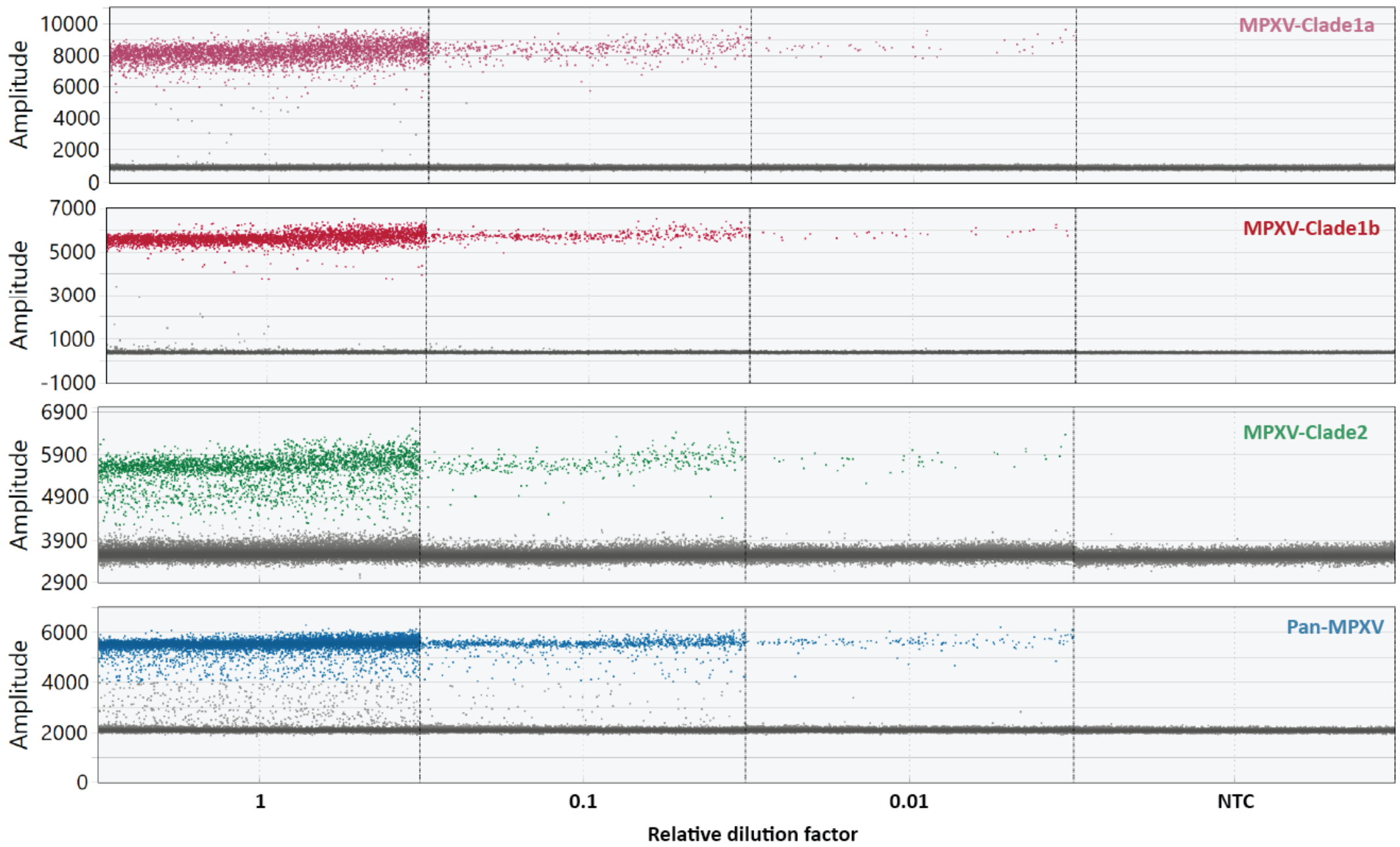

Supplementary Figure S1. One-dimensional visualization of the droplet classification of the clade-specific ddPCR assay targeting MPXV. The horizontal axis represents the tested samples, including a positive control containing all clades at different 10-fold dilutions, as well as a no-template control (NTC). The vertical axis displays fluorescence amplitude. Each droplet is represented by a dark grey dot. Positive droplets are separated by negative droplets by manually setting thresholds that cut midway through the rain. Each panel corresponds to a target detected in a different channel. From top to bottom: MPXV-Clade1a in ROX, MPXV-Clade1b in Cy5, MPXV-Clade2 in HEX, and pan-MPXV in FAM.

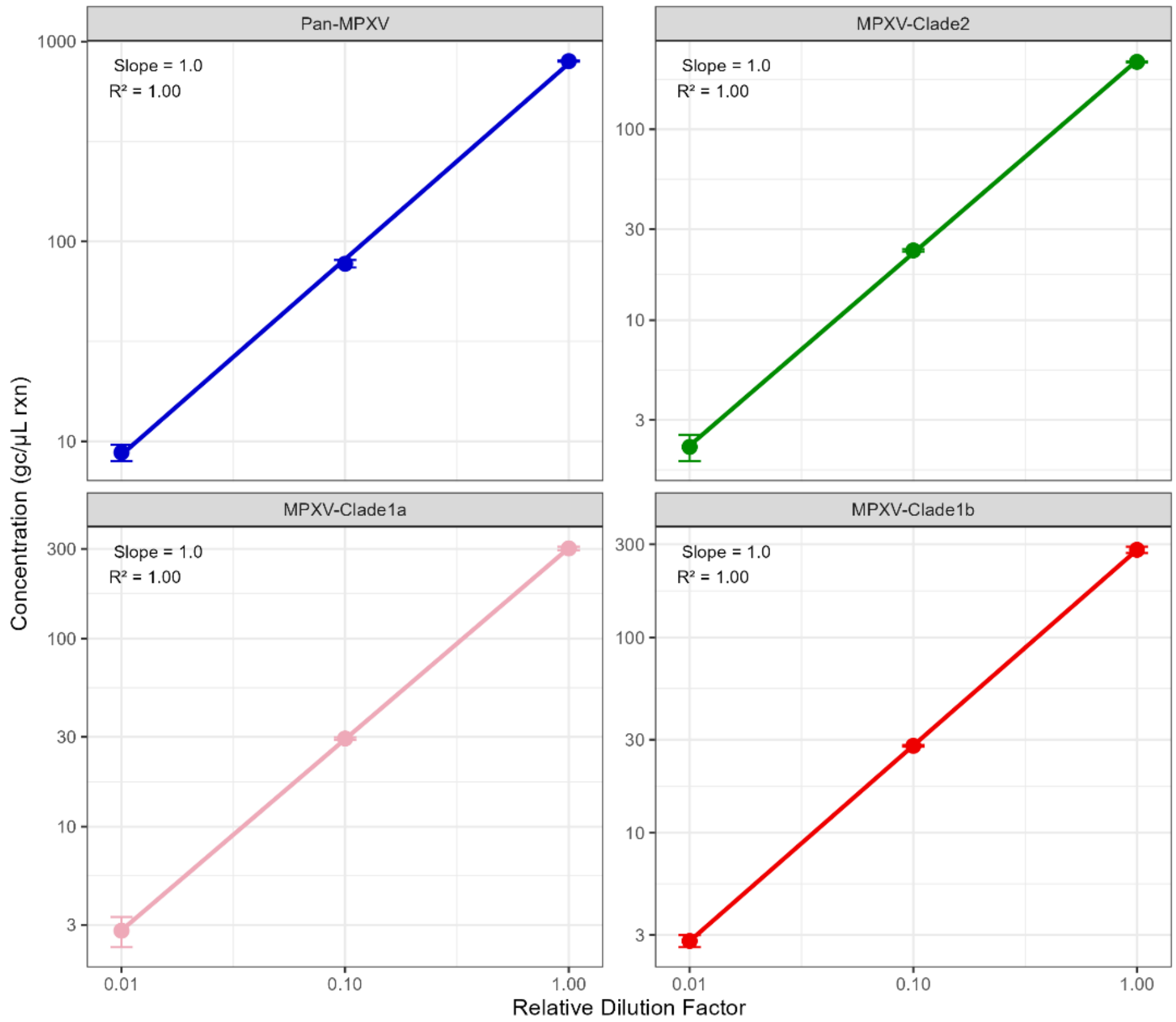

Supplementary Figure S2. Validation of four individual single-plex assays. Each panel represents a different single-plex assay. The vertical axes show the observed concentrations of the targets measured by ddPCR and expressed in genome copies (gc) per microliter (µl) of reaction (rxn). Horizontal axes indicate the relative dilution factors. Linear regression lines were fitted to concentrations and slopes and R^2 values are displayed on each panel. Error bars represent the standard deviation of three technical replicates per measurement.

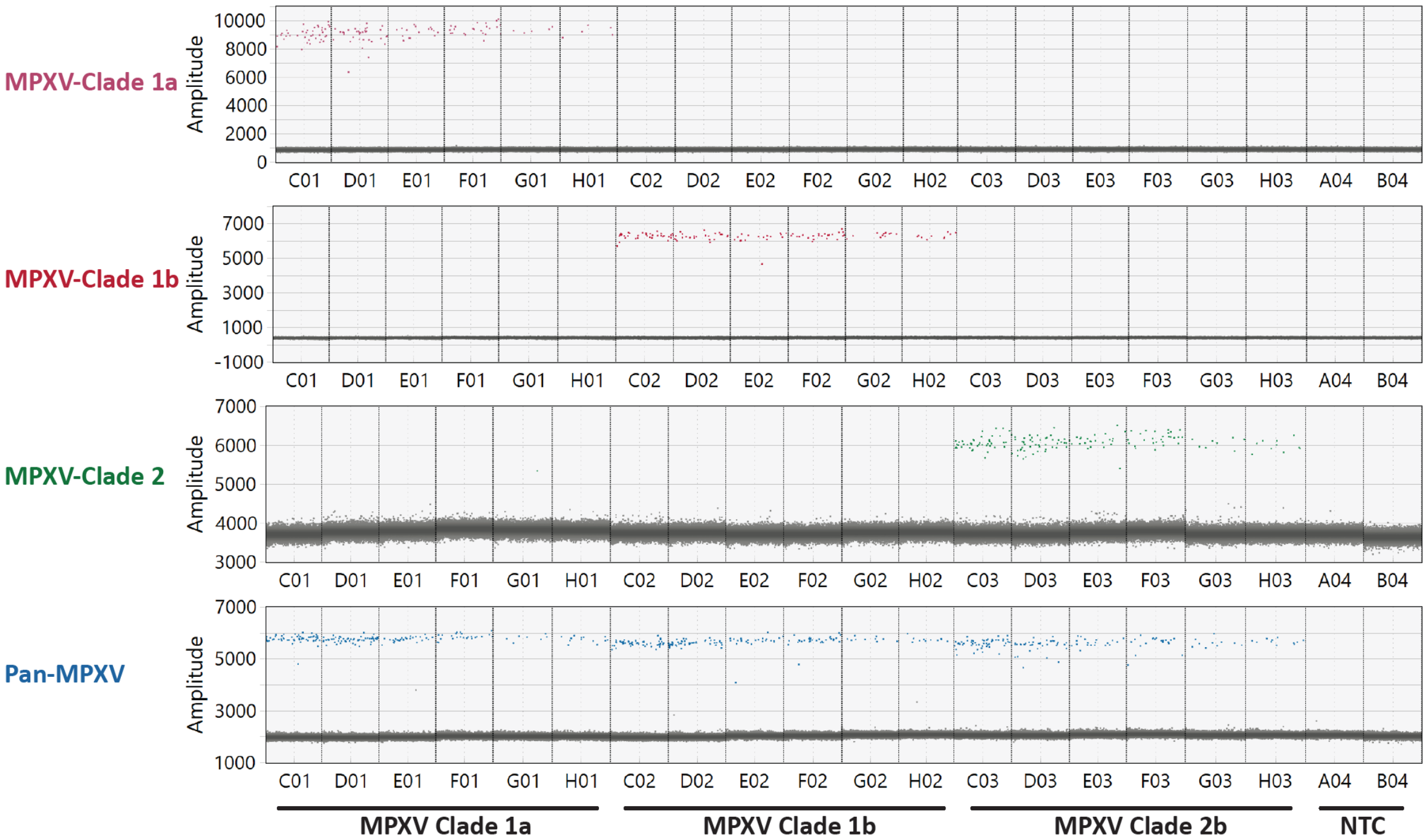

Supplementary Figure S3. Specificity of the four-plex ddPCR assay: testing positive reference samples belonging to different clades. The horizontal axis indicates the tested samples: extracted DNA from clade 1a, clade 1b, and clade 2b, each measured at three different concentrations, as well as a no-template control (NTC). The vertical axis shows the fluorescence amplitude. Each panel corresponds to a target detected in a different channel.

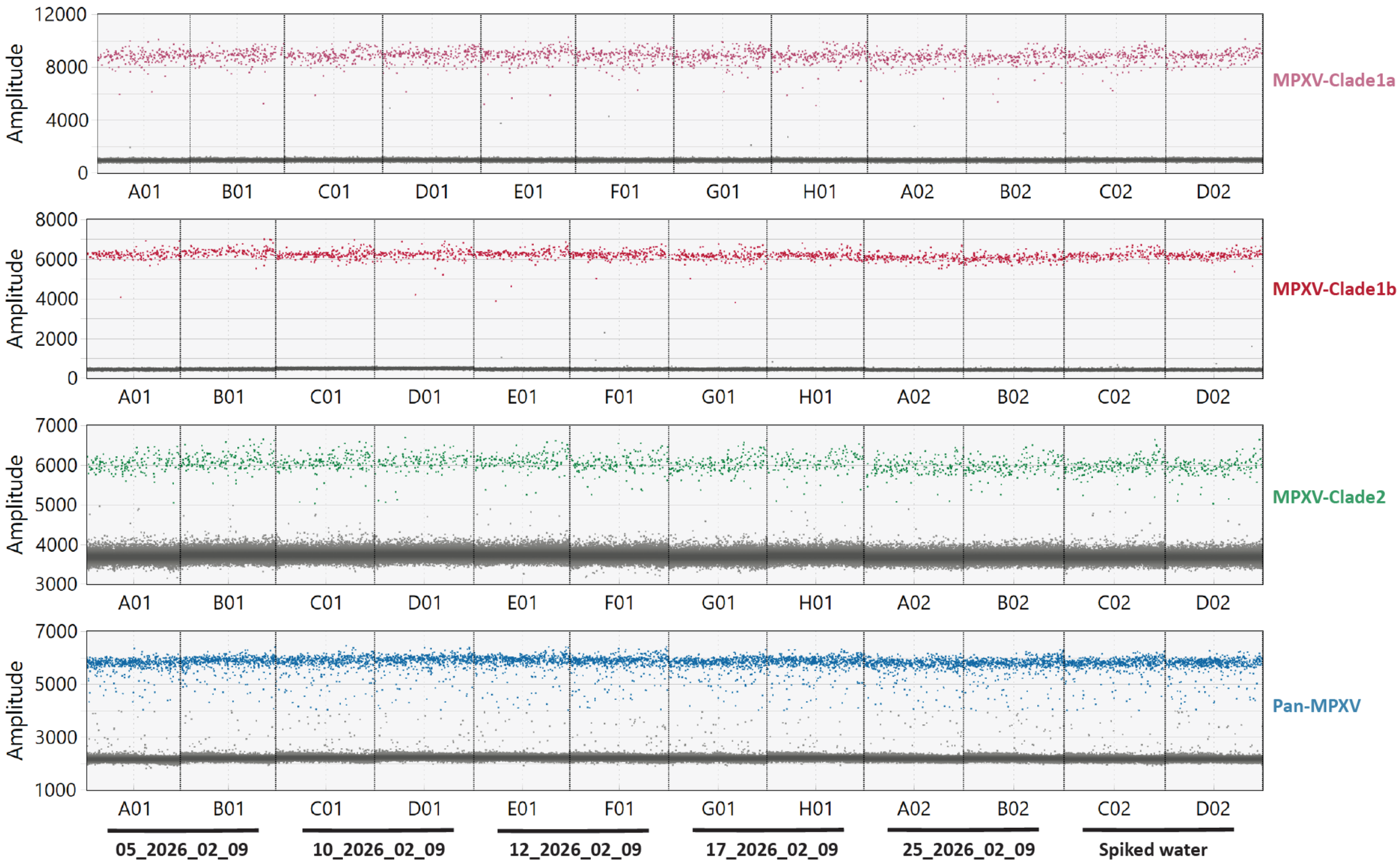

Supplementary Figure S4. MPXV positive material spiked in wastewater extracts. The horizontal axis indicates the tested samples: five different wastewater extracts, each measured in technical duplicates, as well as a spiked water control. The vertical axis shows the fluorescence amplitude. Each panel corresponds to a target detected in a different channel.
